# MOBAS: identification of disease-associated protein subnetworks using modularity-based scoring

**DOI:** 10.1186/s13637-015-0025-6

**Published:** 2015-06-30

**Authors:** Marzieh Ayati, Sinan Erten, Mark R. Chance, Mehmet Koyutürk

**Affiliations:** 1 Department of Electrical Engineering and Computer Science, Case Western Reserve University, 10900 Eucid Ave., Cleveland, 44106 OH USA; 2 Center for Proteomics and Bioinformatics, Case Western Reserve University, 10900 Eucid Ave., Cleveland, 44106 OH USA

**Keywords:** Protein-protein interaction network, Genome-wide association studies, Statistical significance

## Abstract

Network-based analyses are commonly used as powerful tools to interpret the findings of genome-wide association studies (GWAS) in a functional context. In particular, identification of disease-associated functional modules, i.e., highly connected protein-protein interaction (PPI) subnetworks with high aggregate disease association, are shown to be promising in uncovering the functional relationships among genes and proteins associated with diseases. An important issue in this regard is the scoring of subnetworks by integrating two quantities: disease association of individual gene products and network connectivity among proteins. Current scoring schemes either disregard the level of connectivity and focus on the aggregate disease association of connected proteins or use a linear combination of these two quantities. However, such scoring schemes may produce arbitrarily large subnetworks which are often not statistically significant or require tuning of parameters that are used to weigh the contributions of network connectivity and disease association.

Here, we propose a parameter-free scoring scheme that aims to score subnetworks by assessing the disease association of interactions between pairs of gene products. We also incorporate the statistical significance of network connectivity and disease association into the scoring function. We test the proposed scoring scheme on a GWAS dataset for two complex diseases type II diabetes (T2D) and psoriasis (PS). Our results suggest that subnetworks identified by commonly used methods may fail tests of statistical significance after correction for multiple hypothesis testing. In contrast, the proposed scoring scheme yields highly significant subnetworks, which contain biologically relevant proteins that cannot be identified by analysis of genome-wide association data alone. We also show that the proposed scoring scheme identifies subnetworks that are reproducible across different cohorts, and it can robustly recover relevant subnetworks at lower sampling rates.

## Introduction^1^

In recent years, there has been an explosion in genome-wide association studies (GWAS) of complex diseases [1]. These studies have successfully revealed many genetic variants conferring susceptibility to disease. However, GWAS have so far explained a small fraction of the heritability of common diseases and provided limited insights into their molecular mechanisms. A commonly cited reason underlying the limitations of GWAS is the complex nature of diseases, i.e., the interplay among multiple genetic variants in driving disease phenotype. Therefore, many computational methods have been developed to integrate the outcome of GWAS and with other biological such as pathways, annotations, and networks to provide a functional context for disease association of multiple genetic variants [2–5].

Among computational methods that aim to identify multiple genetic variants associated with diseases, identification of disease-associated functional modules has been commonly used as a powerful tool to gain insights into the system biology of disease mechanisms [3]. These methods aim to identify highly connected subnetworks of the human protein-protein interaction (PPI) network (hence, functional module) that exhibit high aggregate association with the disease as indicated by the GWAS p values of associated genetic variants (hence, disease-associated).

These methods have been shown to be effective in uncovering the functional relationships among disease-associated genetic variants for a number of complex diseases, including multiple sclerosis [2], breast cancer and pancreatic cancer [3], and sleep apnea [6]. Meanwhile, Vandin et al. proposed a method called HotNet [7] which uses the mutation data to identify subnetworks in which genes are mutated in a significant number of patients. They are using a diffusion process in a PPI network to identify the significant subnetworks of fixed size which are covering a maximum number of disease cases. However, tools like HotNet cannot be directly applied to GWAS data since the mathematical representation of somatic mutations cannot be directly translated to the representation of case-control differences in germline polymorphisms.

This problem can be considered a generalization of community detection. Community detection is a well-studied problem in network analysis [8–10], and it closely relates to the graph partitioning problem [11–13]. In graph partitioning, the objective is to assign each node to a part such that the edges that are across the parts are minimized. In our problem, however, the focus is to find subnetworks that are high-scoring, and many of the nodes in the network may not be assigned to any subnetwork. In the identification of disease-associated functional modules, a key challenge is to define a scoring function that will accurately assess the “interestingness” of a given subnetwork in terms of functional modularity (network connectivity) and disease association. Note that the aim is scoring individual subnetworks locally rather than globally scoring a partitioning of the network.

While scoring subnetworks, many of the existing methods ignore the degree of network connectivity and score connected subnetworks of the human PPI network using an aggregate of the disease association of comprising gene products [3, 14, 15]. Alternately, some methods incorporate network connectivity by using a linear combination of this aggregate score and the density of the induced subnetwork, using a free parameter to adjust the relative contributions of disease association and network connectivity [16, 17]. Subsequently, they identify high-scoring subnetworks using various algorithmic techniques [15, 16] and empirically assess the significance of these subnetworks based on permutation tests [2].

Scoring schemes that are based on an aggregate of individual disease association scores are highly influenced by subnetwork size, i.e., the number of proteins in the subnetwork. Indeed, it has been observed that existing scoring schemes (e.g., the NODE-BASED scoring scheme implemented in jActiveModules [15]) produce large subnetworks (containing hundreds of proteins), which require further computational analyses for the extraction of their biologically relevant parts [6]. Furthermore, Baranzini et al. [2] systematically show that if correction for multiple hypothesis testing is handled properly, such scoring schemes do not yield statistically significant subnetworks for many diseases. Scoring schemes that incorporate the degree of network connectivity, on the other hand, require tuning of a free parameter to adjust the relative contributions of disease association and network connectivity, making it difficult to apply these algorithms to cases where no training data is available. However, this is the case for many applications since biologically relevant subnetworks for many diseases are largely unknown.

In this paper, we propose a scoring scheme that (i) integrates disease association and network connectivity in a parameter-free fashion and (ii) incorporates an approximation of the statistical significance of this integrated score. The key idea of the proposed method is to assess the disease association of each interaction in the network and account for the background disease association as an approximation to statistical significance. In this respect, the proposed approach may be thought of a generalization of Newman’s [18] measure of modularity, which was developed for community detection in networks.

We test the proposed scoring scheme on GWAS data for type II diabetes (T2D) and psoriasis (PS). We use the T2D dataset to compare the performance of the proposed method with two most commonly used scoring methods. Then, we use two independent PS datasets to investigate the reproducibility of the identified subnetworks across different cohorts.

Our results show that subnetworks that are scored highly by the proposed scoring scheme are more likely to be statistically significant as compared to those that are scored high by the other two scoring schemes. We also assess the biological relevance of identified subnetworks in terms of their inclusion of known disease-related proteins that do not exhibit significant disease association based on individual analysis of GWAS data. Our results suggest that the proposed scheme yields parsimonious subnetworks that contain known proteins, as well as those that are not individually significant, but are candidates for further investigation. Moreover, our results show that the identified subnetworks are robust at lower number of samples. We also investigate the reproducibility of the subnetworks across different cohorts.

## Methods

In this section, we first describe the problem setting for the identification of disease-associated functional modules. Then, we describe the three scoring schemes we consider in this study. Subsequently, we describe the algorithms used to identify high-scoring modules according to this scoring scheme. Finally, we discuss our methodology for assessing the statistical significance of identified high-scoring modules.

### Problem setting

The input to the problem of identifying disease-associated functional modules (DAFM) is a graph *G*=(*V*,*E*,*w*) that represents the human PPI network. Here, *V* denotes the set of proteins, *E* denotes the set of pairwise interactions between these proteins, and $w:E \rightarrow \mathbb {R}$ denotes edge weights, where *w*(*u*,*v*) represents the likelihood that proteins *u*,*v*∈*V* interact. The likelihood scores for interactions are usually computed by integrating the outcome of several experimental and computational methods for detecting and predicting protein-protein interactions. In this paper, we use an online tool, MAGNET [19], to score the interactions.

Besides the network, we are given a genome-wide association (GWAS) dataset *D*=(*C*,*M*,*g*,*f*), where *C* denotes the set of genomic loci that are assayed, *M* denotes the set of samples, *g*(*c*,*m*) denotes the genotype of locus *c*∈*C* in sample *m*∈*M*, and *f*(*m*) denotes the phenotype of sample *m*∈*M*. If the phenotype is dichotomous (i.e., *f*:*M*→{0,1} where 1 denotes case and 0 denotes control), then the disease association of each variant is computed using the standard statistical test, e.g. Cochran-Armitage trend test, Fisher’s exact test, or Cochran-Mantel-Haenszel tests [20]. For quantitative traits (i.e., $f: M \rightarrow \mathbb {R}$), association tests such as Breslow-Day or homogeneity of odds ratio are common to use [21].

In this paper, our focus is not on assessing the disease association of each variant. We rather assume that the statistical significance of the association of each locus *c*∈*C* with the disease is given as a p value, denoted *p*(*c*). From these significance values, we compute the significance of the association of each gene coding for a protein *v*∈*V* by taking the most significant association of the variants that lie within the region of interest for that gene. For the experiments reported in this paper, we define the region of interest for a gene, denoted *N*(*v*)⊂*C*, as the genomic region within 20 kb up- and downstream the coding region for the gene. We further log-transform the significance of disease association for each gene *v*∈*V* to obtain disease association score

(1) $$ r_{v}=\max_{c \in N(v)}\{-\log(p(c))\}.   $$

The objective of the disease-associated functional module (DAFM) identification problem is to identify PPI subnetworks such that:

- the subnetwork is enriched in proteins that are associated with the disease,
- the proteins in the subnetwork are functionally associated with each other.

Consideration of these two criteria together enables identification of functionally modular processes that are associated with the disease. An important challenge in this regard is to develop scoring schemes to achieve a reasonable balance between these two criteria so that the subnetworks that are assigned statistically significant scores are those that are biologically most meaningful and useful.

### Scoring subnetworks

**Fig. 1:**
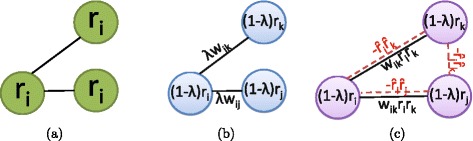
Illustration of existing and proposed scoring schemes. This figure shows the scoring schema for quantifying the disease association of protein subnetworks: **a** NODE-BASED scoring, **b** LINEAR COMBINATION of node scores and edge scores, **c** the proposed MODULARITY-BASED (MOBAS) scoring scheme. For each method, the score of subnetwork is computed as an aggregate of all quantities in the figure

In this section, we describe the three scoring schemes that are used in our experimental studies. These scoring schemes are illustrated in Fig. 1. Two of these schemes are based on existing methods for the identification of active subnetworks using gene expression data, and these methods are commonly used in integrating GWAS outcome with PPI networks. The third is a novel scoring method that is based on a measure of modularity in networks [10].

Node-based scoring: A popular method for scoring subnetworks is implemented in JActiveModules [15], a Cytoscape plug-in for the identification of “active subnetworks”. This method was originally designed to integrate gene expression data with PPI networks to identify PPI subnetworks that are differentially expressed (or “active”) under a certain condition or phenotype. However, it has also found common application in integrating GWAS data with PPI networks to identify disease-associated subnetworks. It takes as input the individual disease association scores for all proteins and aims to identify connected subgraphs of the PPI network with high aggregate association score. More precisely, under this scoring scheme, a subnetwork *Q*⊂*V* that induces a connected subgraph in the PPI network is scored as follows:

(2) $$ \sigma_{N}(Q) = \frac{1}{\sqrt{|{Q}|}}\sum_{u\in Q}{r_u}.  $$

The NODE-BASED scoring scheme is illustrated in Fig. 1 a. Since this scoring scheme is based on the individual disease association of the proteins composing the subnetwork, we refer to it as NODE-BASED scoring. Under this scheme, the connectivity of the subnetwork is imposed as a qualitative constraint to ensure that the proteins in the subnetwork are functionally related. However, the degree of connectivity, hence the degree of functional association among the proteins, is not quantified.

Many studies in the context of a related problem, candidate disease gene prioritization, have shown that the degree of network connectivity provides valuable information on the functional relationships between individual proteins in terms of their association with disease [22, 23]. To this end, taking into account the degree of network connectivity may lead to the identification of more relevant networks since it may better account for the noise in the network, as well as the modularity of the processes in which the proteins are involved together.

Linear combination of node and edge scores : Disease association and the degree of connectivity in the network are two criteria that are not readily comparable. Therefore, incorporation of these two criteria into a single scoring scheme is rather challenging. To this end, scoring subnetworks based on a linear combination of the two criteria is reasonable, in that it provides a framework in which the relative contributions of the two criteria are adjusted using a single parameter. Motivated by this observation, Ma et al. [16] propose the following LINEAR COMBINATION-based scoring scheme for the identification of disease-associated subnetworks:

(3) $$ \sigma_{L}(Q) = \lambda \frac{\sum_{u,v \in Q}{w(u, v)}}{\sqrt{{|Q|}\choose{2}}} + (1-\lambda) \frac{\sum_{u\in Q}{r_u}}{\sqrt{|Q|}}.  $$

This approach has been shown to be more effective than NODE-BASED scoring in the context of identifying “active subnetworks” using gene expression data [16]. However, to the best of our knowledge, it has not found application in the identification of disease-associated subnetworks using GWAS data. An important drawback of this approach is its dependence on a tunable parameter, since the objective of DAFM is to find subnetworks that exhibit statistically significant association with the disease in an unsupervised manner, and training data (i.e., “known” disease-associated subnetworks) are rarely available.

Modularity-based scoring (MOBAS): The objective in any pattern discovery problem for biological applications is to discover patterns that are *statistically significant*. To this end, it is important to note that “high scoring” does not necessarily mean statistically significant and a scoring scheme should not be overly conservative or overly relaxed, since a conservative scoring scheme may not produce any non-trivial high-scoring patterns and a relaxed scoring scheme may produce high scoring patterns that are not significant. Here, we argue (and show in Section 2) that both NODE-BASED and LINEAR-COMBINATION-based scoring schemes are overly relaxed in that they lead to the identification of very large subnetworks that will achieve high scores just because of their size, since these scoring schemes do not explicitly penalize for the inclusion of more proteins in the subnetwork.

We here propose a novel scoring scheme that integrates degree of network connectivity with disease association in a parameter-free manner by assessing the disease association of each pair of proteins (a potential interaction) in the network. Further, building on Newman’s [10] measure of modularity for community detection in networks, the proposed scoring scheme incorporates an approximation of statistical significance into the scoring of subnetworks by taking into account the background disease association scores.

We define the disease association of a pair of proteins *u*,*v*∈*V* as follows:

(4) $$ s_{uv} =\left\{ \begin{array}{ll} w(u,v) r_{u} r_{v} & \text{if} \ uv \in E \\ 0 & \text{otherwise} \end{array}\right.  $$

Recall that *r*_*u*_ indicates the likelihood that protein *u* is associated with the disease of interest. Therefore, *s*_*uv*_ provides a measure of the disease association of the interaction between *u* and *v* with the disease;

We then define the disease association score of a given subnetwork *Q*⊆*V* as follows:

(5) $$ \sigma_{M}(Q) = \sum_{u,v\in Q}{s_{uv}-\hat{r}_{uv}},  $$

where $\hat {r}_{\textit {uv}}$ denotes the “background” disease association score of the interaction of proteins *u* and *v*.

In other words, the disease association of subnetwork *Q*⊆*V* is defined as the linear combination of the differences between the observed and background disease association scores of all potential pairwise interactions in the subnetwork. Note that, it is assumed that an interaction exists between every pair of proteins in the background, therefore any pair of proteins in the subnetwork that do not interact with each other are penalized by a factor of their background interaction association scores. For this reason, groups of proteins that induce a heavily connected subgraph in the PPI network are favored by this scoring scheme. Note that, to account for the variance in disease association scores and network connectivity, it would be more informative to normalize *σ*_*M*_ by the standard deviation of the respective variables. However, because of the dependencies in different variables, this would complicate the definition and the computation of the scoring function considerably. For this reason, we formulate *σ*_*M*_ in a simple form while still incorporating the background expectations.

To avoid making assumptions on the distribution of disease association scores, we compute these background scores empirically for each protein pair. For this purpose, we randomize the original GWAS data by permuting the labels of the samples to break the relationship between the genotype and phenotype, while preserving the distribution of genotypes for each locus and also preserving the relationship between loci. We repeat the permutation multiple (*N*) times such that the number of samples derived from the distribution is sufficiently large and the computation is feasible (we use *N*=100 in our experiments). For each randomized instance 1≤*i*≤*N*, we compute the disease association of gene *u* on that instance *i* as $r_{u}^{(i)}$ using Eq. 1. Subsequently, we compute the background disease association of interaction between each gene pair of *u* and *v* as

(6) $$ \hat{r}_{uv}=\sum_{i=1}^{N}{\left(r_{u}^{(i)}\times r_{v}^{(i)}\right)}/N.  $$

### Searching for high scoring subnetworks

Subnetwork search queries with combinatorial objective functions often lead to NP-hard problems. For this reason, existing methods for identifying disease-associated functional modules use approximation algorithms or heuristics, such as greedy algorithms, simulated annealing [15], genetic algorithms [16], or linear programming based on a continuous approximation [17]. Since our focus here is on the development of a sound scoring function, the algorithm we use to search for high scoring subnetworks should be compatible with those implemented by existing methods, so that the scoring functions can be compared without any algorithmic bias.

Here, for simplicity, we implement a greedy algorithm as well. Namely, to find all high-scoring subnetworks, we search the PPI network by starting from the protein with most significant disease association, repeatedly examining the proteins in the neighborhood of the proteins so far in the subnetwork, and adding to the subnetwork the protein that provides the best improvement of the subnetwork score. We repeat these steps until we cannot find any neighboring protein that improves the subnetwork score. We further reduce the computational complexity of the search algorithm by constraining the search space to a locality in the network (i.e., within two jumps of the first protein added to the subnetwork). Once a subnetwork with maximal score is found, we save it as a high-scoring subnetwork and remove its constituent proteins from the network. We then repeat the procedure to find other high scoring modules, until the entire network is exhausted. Finally, we sort all identified modules according to their score and assess the statistical significance of their scores.

### Assessment of statistical significance

The proposed scoring scheme approximates the statistical significance of subnetworks by accounting for the background distribution of disease association. However, the distributions used in this approximation do not take into account multiple hypothesis testing, since each subnetwork is scored independently. Furthermore, only sample means are incorporated in the scoring function, which may not account for the variability in the distributions of network connectivity and disease association. Consequently, high-scoring modules identified using the proposed scoring scheme are not necessarily significant. For this reason, for all the three scoring schemes that are considered, we assess the statistical significance of all identified subnetworks using empirical distributions generated by running the algorithm on multiple randomized datasets.

We generate the randomized datasets using two different approaches:

1. Random permutation of the phenotypes of samples, with a view to testing the hypothesis that the high score of each identified subnetwork arises from the correlations between genotype and phenotype.
2. Random permutation of the PPI network while preserving the degree distribution, with a view to testing the hypothesis that each high-scoring subnetwork is composed of functionally associated proteins. To generate random networks with the same degree distribution as the original network, we repeatedly swap interactions between randomly chosen pairs of proteins [24].

Observe that, since the number of hypotheses being tested is equal to the number of potential connected subnetworks of the PPI network, multiple hypothesis testing poses an important challenge in evaluating the significance of identified subnetworks. We tackle this challenge by using the ranking of subnetworks identified on random datasets to generate a null distribution for each subnetwork based on its rank on the original dataset. Namely, for the subnetwork that has the *i*th highest score on the original dataset, we test the hypothesis that the algorithm could discover at least *i* subnetworks with higher or equal score even if the phenotypes and the interactions in the network were assigned at random.

To be more precise, we generate a sufficiently large number (*M*) of randomized datasets for each type of permutation (i.e., randomized genotype and randomized PPI). Then, we identify and rank all high-scoring subnetworks on each dataset. We then assess the statistical significance of each subnetwork identified on the original data by comparing its score against the scores of the subnetworks that are ranked at least as high as itself on the randomized datasets. Namely, for subnetwork *Q*_*i*_ that is ranked *i*th in the original dataset, we take the highest scoring *i* subnetworks from each of the *M* datasets and compute the fraction of subnetworks among these *Mi* subnetworks whose scores are at least as high as that of *Q*_*i*_. We call this fraction the *q* value of the subnetwork, since it implicitly accounts for multiple hypothesis testing.

We call a subnetwork is significant only if its *q* value for both types of permutation is below a preset *q* value threshold. We use *M*=100 (as a trade-off between feasibility and statistical power) and a *q* value threshold of 0.05 in our experiments.

### Software description

MOBAS is implemented in Java and provided in the public domain (http://compbio.case.edu/mobas/) as an open source software. It provides a simple and and easy-to-use graphical user interface. The software requires four input files:

- SNP association file (.assoc) which contains the p value of each SNP with phenotype.
- Mapping file which specifies the mapping of each SNP to one or more genes.
- PPI file which represents the protein protein interaction network. The algorithm is designed to work with a weighted PPI network, which can also be used to work with unweighted networks.
- A set of SNP association files which provide the background distribution of individual associations for SNPs. These files have to be generated by permuting the label of samples in genome-wide association data (Since some GWAS pose limitations on data sharing, we do not provide the permutation service).

MOBAS returns its output as a file that contains the rank, size, score, and the genes of all the identified subnetworks. The user can analyze and visualize the subnetworks using the source code provided on the website.

## Results

In order to test the performance of MOBAS, we use multiple datasets for two different complex diseases: type II diabetes (T2D) and psoriasis (PS). Since we have GWAS data for a single cohort for T2D, we use T2D dataset to investigate the statistical significance of the subnetworks identified by the proposed scoring scheme and compare these with those identified by aggregation of node scores (NODE-BASED) and linear combination of node and edge scores (LINEAR COMBINATION). Then, we use two independent datasets for psoriasis to assess the statistical significance of the subnetworks identified by the proposed scoring scheme and investigate the reproducibility and robustness of the proposed method.

*Association analysis for individual SNPs*: We compute the statistical significance of the association of each SNP with disease using PLINK [25], a well-established toolkit for whole-genome association analysis.

*SNP-gene mapping and association analysis for individual genes*: To compute the disease-association for individual genes, we map SNPs to genes by defining the region of interest (ROI) for a gene as the genomic region that extends from 20 kb upstream to 20 kb downstream of the coding region for that gene. We compute the disease association of each gene as the minimum of the *p* values of the SNPs in the region of interest for that gene, that is the *p* value of the most significant SNP associated with the gene. We log-transform these values to obtain a disease association score for each gene.

*Protein-protein interaction (PPI) dataset*: We use a comprehensive human PPI network downloaded from NCBI Entrez Gene Database [26]. This database integrates interaction data from several PPI databases, including HPRD, BioGrid, and BIND. The PPI network contains 56,110 interactions among 7692 proteins. We assess the reliability of each interaction in this dataset using MAGNET [19], a web service that uses logistic regression to assign reliability scores to PPIs.

*Biological relevance assessment:* We assess the biological relevance of the identified subnetworks using a literature-driven list of genes and processes that have been reported to be associated with diseases. We also perform pathway enrichment analysis to identify the biological processes and pathways potentially associated with diseases. We also investigate the biological relevance of the “novel genes” identified by the scoring gene, namely those that are not known to be associated with the disease, do not show significant disease association according to GWAS data, but are recruited in the significant subnetworks identified by the proposed scoring scheme.

### Results on type II diabetes data

*GWAS dataset*: To evaluate the performance of the proposed method, we use a type II diabetes (T2D) case-control dataset, obtained from Wellcome Trust Case-Control Consortium (WTCCC) [27]. The T2D data contains SNP microarray data for 500,000 SNPs on 1999 case and 1504 control samples (1958 British Birth Cohort). For this dataset, we use the genotype calls provided by WTCCC, which were obtained by using CHIAMO. SNPs with > 10 *%* missing genotypes are excluded from the analyses.

*Genes reported to be associated with T2D*: In order to assess the biological relevance of identified subnetworks, we use a manually curated database of genes that are reported to be associated with T2D in the literature [28]. This list contains 286 genes. We also use a second database that is generated by using seven independent computational disease gene prioritization methods [29], namely GeneSeeker [30], POCUS [31], G2D [32], PROSPECTR [33], eVOC annotation [34], DGP [35], and SUSPECTS [36].

*Pathway enrichment analysis*: We also evaluate the subnetworks that are found to be significantly associated with T2D using pathway enrichment analysis. For this purpose, we use Ingenuity Pathway Analysis (IPA), a commercial software that uses a manually curated and highly reliable database of pathway associations to perform pathway enrichment analysis.

#### Significance of identified subnetworks

In this section, we investigate the statistical significance of the subnetworks identified by each scoring scheme. For this purpose, we compare the scores of highest scoring subnetworks identified on the WTCCC dataset with that of the highest scoring subnetworks identified on 100 randomized datasets in which (i) the sample phenotypes are permuted and (ii) PPIs are randomly permuted while preserving the number of interactions for each protein.

**Fig. 2:**
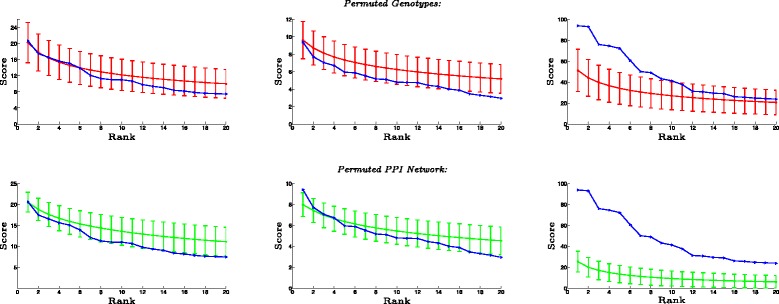
Statistical significance of subnetworks. Statistical significance of high-scoring subnetworks identified using NODE-BASED scoring (*first column*), LINEAR COMBINATION of node scores and edge scores (*second column*), and MODULARITY-BASED (MOBAS) scoring (*third column*). The highest scoring 20 subnetworks identified using each scoring scheme are shown. The *x-axis* shows the rank of each subnetwork according to their score, and the *y-axis* shows its score. The *blue curve* shows the scores of the subnetworks identified on the WTCCC-T2D dataset. For each *i* on the *x-axis*, the *red* (*green*) *curve* and *error bar* in the *first* (*second*) *row* show the distribution of the scores of *i* highest scoring subnetworks in 100 datasets obtained by permuting the genotypes of the samples (permuting the interactions in the PPI networks while preserving node degrees)

The results of this analysis are shown in Fig. 2.

The null distribution displayed in Fig. 2 is precisely the distribution used to compute the *q* values of each identified subnetworks, as described in Section 2.

As seen in top row of Fig. 2, the nine highest scoring subnetworks identified using MOBAS have scores at least one standard deviation above the mean of the top subnetworks identified on randomized datasets. At a q value threshold of 0.05, two of these subnetworks are detected to be statistically significant. In contrast, all subnetworks identified by LINEAR COMBINATION and NODE-BASED scoring are within one standard deviation of the average score of the top subnetworks identified on randomized datasets. In other words, when the existing genotype-phenotype relationship in the dataset is broken via randomization of samples, LINEAR COMBINATION and NODE-BASED still detect subnetworks that score high. The respective *q* values are shown in Table 1.

**Table 1:** Statistical significance of top two subnetworks. The table shows the *q* value of top two subnetworks identified using each scoring scheme according to the permuted genotype and PPI for WTCCC-T2D

Scoring method	Size	*q* value in	*q* value in
		permuted genotype	permuted PPI networks
NODE-BASED	187	0.37	0.45
	190	0.70	0.92
LINEAR COMBINATION	41	0.46	0.09
	17	0.79	0.52
MOBAS	14	0.04	< 0.01
	14	0.05	<0.01

We observe a similar pattern when we compare the subnetworks identified on the original data to those identified on randomly permuted PPI networks.

Baranzini *et al.* [2] also investigate this issue systematically on a number of complex diseases and show that, while the subnetworks identified by jActiveModules (NODE-BASED scoring) on some diseases (including multiple sclerosis and rheumatoid arthritis) are significant, many subnetworks that are identified for other diseases are not, including those for T2D. Our results stand as a reproduction of these results and suggest that the proposed modularity-based scoring scheme does not suffer from this problem.

To choose significant subnetworks for further investigation, we require statistical significance in terms of both disease association and network connectivity. For this purpose, we compute the *q* value of each subnetwork as the maximum of its *q* values with respect to permuted genotype and permuted PPI. Consequently, only the two subnetworks identified by the proposed method are deemed statistically significant at a false discovery rate of *q*<0.05.

#### Biological relevance

**Fig. 3:**
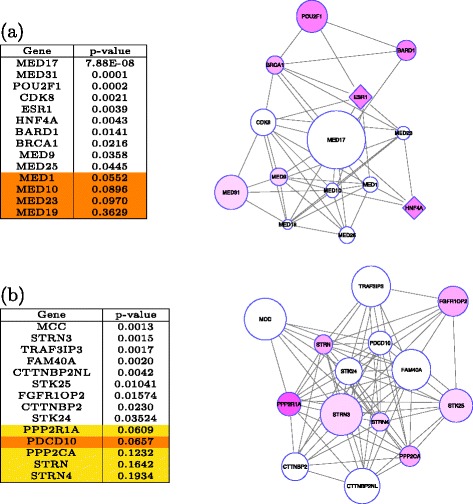
Two significant subnetworks. **a** Top subnetwork and **b** second top subnetwork that are found to be significantly associated with T2D. The size of each node indicates the significance of the association of the corresponding protein with T2D (*r*_*v*_). The *diamond nodes* are those previously reported to be associated with T2D in the literature [28]. The intensity of *purple coloring* in the nodes indicates the number of computational disease gene prioritization methods [29] that identified the respective gene to be associated with T2D. The individual *p* values of each gene in the subnetwork are shown in the table left of the subnetwork. The genes with insignificant *p* value (*p*>0.05) that are known to be related to T2D are highlighted in *yellow*. The genes with insignificant p value and are not reported to be related to T2D are highlighted in *orange*. These genes are the candidates for further investigation

In this section, we investigate the biological relevance of the two statistically significant subnetworks (*q*<0.05) identified by the proposed method. These two subnetworks are shown in Fig. 3. According to Ingenuity Pathway Analysis (IPA) software, the top subnetwork (Fig. 3 a) is significantly enriched in Estrogen Receptor Signaling (*p*<3.42*E*−12) and Glucocorticoid Receptor Signaling (*p*<1.19*E*−3). The second subnetwork (Fig. 3 b) is significantly enriched in Wnt/ *β*-catenin Signaling (*p*<0.01) and Cell Cycle Regulation by BTG Family Proteins (*p*<2.2*E*−4).

The association between a region of the estrogen receptor- *α* (ESR1) gene and T2D is reported in the literature [37]. Although the *p* value of its association with T2D according to GWAS data before correction for multiple hypotheses is moderate (*p*<0.003), this gene appears in the most significant subnetwork identified by the proposed algorithm. This subnetwork is significantly enriched in estrogen receptor signaling pathway, which is known to play a crucial role on insulin resistance syndrome [38]. Glucocorticoid excess in vivo has been shown to cause decreased insulin sensitivity and insulin receptor binding in target tissues [39]. The first subnetwork is also enriched in glucocorticoid receptor signaling. As shown in Fig. 3 a, this subnetwork contains nine subunits of mediator complex which has an important role in regulating lipid metabolism linked to major human diseases including type II diabetes [40].

The second subnetwork is enriched in Wnt/ *β*-catenin signalling, which is a well-known pathway related to T2D. STRN, STRN4, and PPP2CA are previously reported to be associated with T2D but do not have significant *p* value according to the association analysis for individual variants (respectively 0.16, 0.19, and 0.12 before correction for multiple hypothesis testing). The subnetwork discovered using the proposed scoring scheme reveals the involvement of these genes in T2D-related processes, demonstrating that network analysis provides information beyond what GWAS would detect alone.

### Results on psoriasis data

In order to investigate the generalizability of the results obtained on T2D to a broader set of diseases, we also perform a similar analysis on psoriasis, a complex auto-immune disease, for which some genomic factors with strong association have been already identified (e.g., HLA) [41].

*GWAS datasets*: We use two GWAS datasets for psoriasis, one obtained from the database of Genotypes and Phenotypes (dbGaP) within the framework of the Genetic Association Information Network (GAIN) [42] and the other obtained from the Wellcome-Trust Case Control Consortium (WTCCC) [43]. GAIN genotyped 438,670 SNPs in 1409 European ancestry psoriasis cases and 1436 controls. However, a collection of 950 cases and 692 controls designated as appropriate for general research use. WTCCC data has 594,224 SNPs in 2178 case samples recruited from five centers in England, Scotland, and Ireland, and WTCCC control samples include 2501 healthy blood donors from the United Kingdom Blood Service (UKBS) collection and 2674 individuals from the 1958 Birth Cohort (58C) dataset. Since we are interested in assessing the reproducibility of identified subnetworks, we work on the genomic loci for which genotype information is available in both data sets. We filter out the SNPs with MAF greater than 5 %. The two datasets have genotype information for 146,213 SNPs in common.

#### Statistical significance

**Fig. 4:**
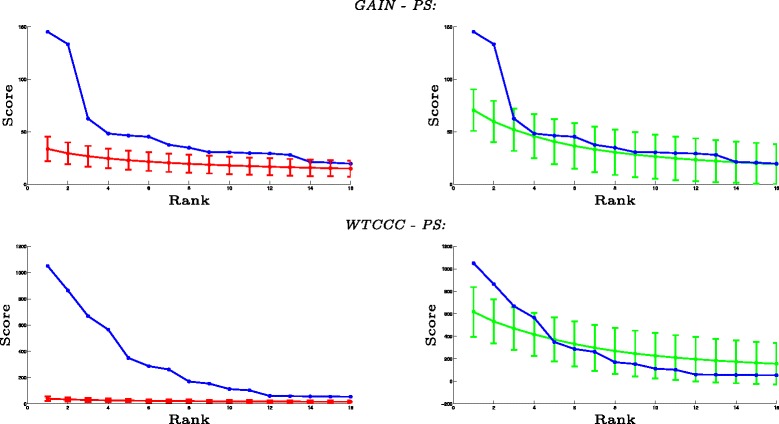
The statistical significance of high-scoring subnetworks using MOBAS on psoriasis dataset. The highest scoring 16 subnetworks identified using MOBAS are shown. The *x-axis* shows the rank of each subnetwork according to their score, and the *y-axis* shows its score. The *first row* shows the result of MOBAS on GAIN–PS dataset, and the *second row* shows the result on WTCCC–PS dataset. The *blue curve* shows the scores of the subnetworks identified on the dataset. For each *i* on the *x-axis*, the *red* (*green*) *curve* and *error bar* show the distribution of the scores of *i* highest scoring subnetworks in 100 datasets obtained by permuting the genotypes of the samples (permuting the interactions in the PPI networks while preserving node degrees)

In this section, we investigate the statistical significance of the subnetworks identified by MOBAS. For this purpose, we compare the scores of identified subnetworks with the highest score of subnetworks identified on 100 randomized permutation of samples and also permutation of interactions in PPI network while preserving the degree distribution. Figure 4 shows the result of this analysis. The first row shows the significance of the high scoring subnetworks on the GAIN dataset, and the second row shows the result on WTCCC dataset. As seen in the first row of Fig. 4, the first 13 highest scoring subnetworks identified using MOBAS have scores of at least one standard deviation above the mean of the top subnetworks identified on datasets with randomized phenotype. At a *q* value threshold of 0.05, seven of these subnetworks are detected to be statistically significant. The *q* values are shown in Table 2. Using the same analysis for permuted PPI, we find that the first two subnetworks are statistically significant. The second row of Fig. 4 shows that the first 16 subnetworks have significantly higher scores compared to randomized phenotype, whereas just the first two of them are significant compared to randomized PPI. As demonstrated by these results, the subnetworks discovered on both PS datasets are more significant according to the randomized phenotype model as compared to the randomized network model. This observation is concordant with previously reported observations indicating that there are certain variants with very significant marginal association with PS [42].

**Table 2:** Statistical significance (*q* value) of top subnetworks. The subnetworks are identified using MOBAS according to the permuted genotype and PPI on two independent psoriasis datasets

Rank	GAIN - PS			WTCCC - PS		
	Size	*q* value in	*q* value in	Size	*q* value in	*q* value in
		permuted genotype	permuted PPI networks		permuted genotype	permuted PPI networks
1	13	<0.01	<0.01	15	<0.01	0.05
2	8	<0.01	<0.01	26	<0.01	0.06
3	9	0.01	0.23	10	<0.01	0.12
4	6	0.02	0.38	9	<0.01	0.14
5	9	0.02	0.33	30	<0.01	0.47
6	5	0.02	0.33	5	<0.01	0.51
7	4	0.04	0.33	12	<0.01	0.49

#### Reproducibility across different cohorts

**Fig. 5:**
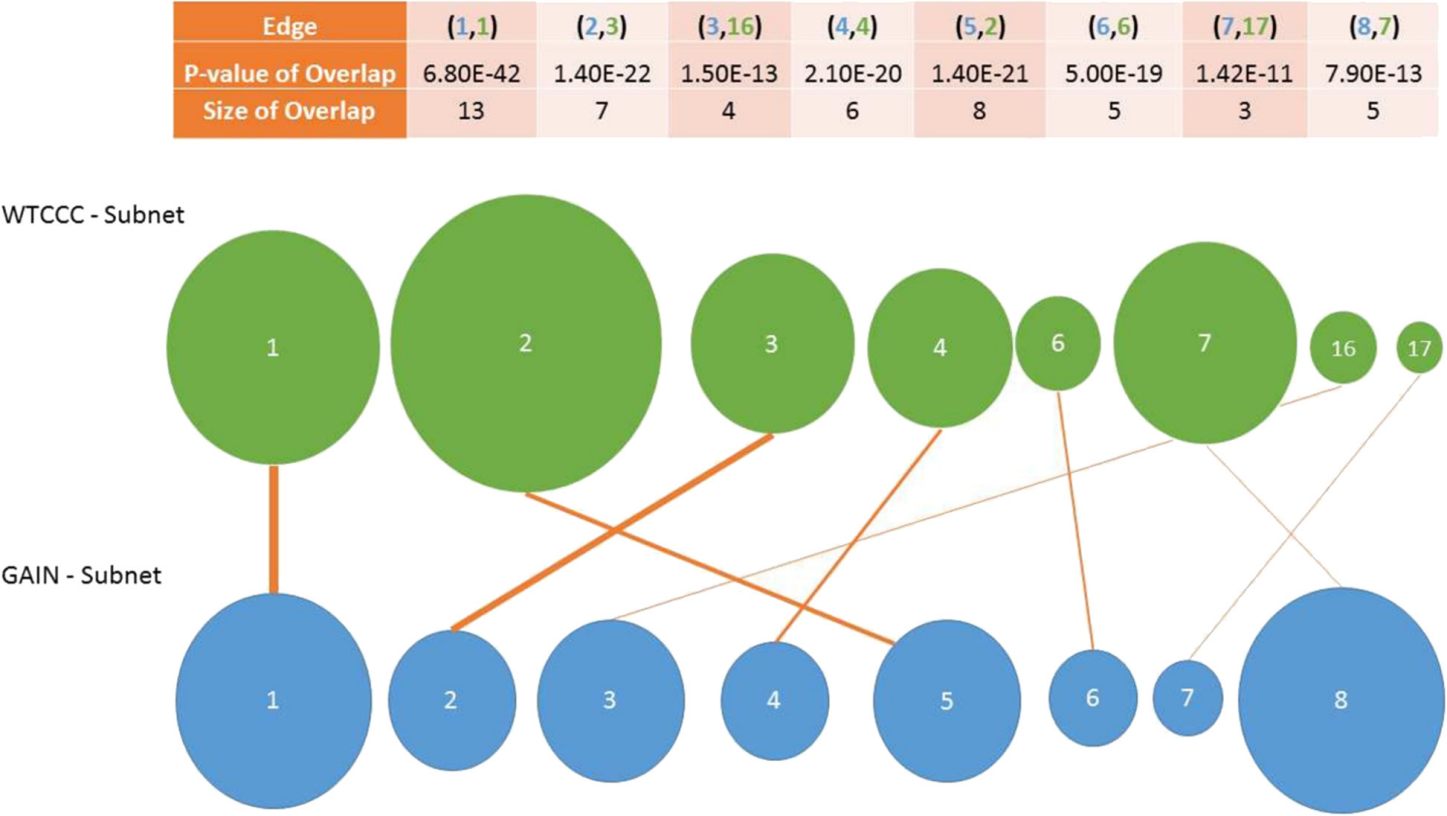
Reproducibility of identified subnetworks using MOBAS in two independent datasets. The size of the circles represents the size of identified subnetwork. The thickness of the edges represents the significance of overlap between the two subnetwork based on hypergeometric distribution

To investigate whether the identified significant subnetworks are reproducible, we assess the overlap between the top subnetworks identified on the two datasets. All the nodes which are in the top subnetwork identified on the GAIN dataset are also included in the top subnetwork identified on the WTCCC dataset. These subnetworks are shown in Fig. 5. In the figure, the green circles represent the subnetworks identified on WTCCC and the blue circles represent the subnetworks identified on the GAIN dataset. The size of each circle represents the size of the subnetwork, and the number in the circle shows the rank of the subnetwork among all subnetworks discovered on the respective dataset. Each edge represents the overlap between two subnetworks, and the thickness of an edge represents Bonferonni-corrected significance of overlap computed based on the hypergeometric distribution. The significant overlap between the top identified subnetworks on the two datasets shows that the identified subnetworks are highly reproducible.

To further investigate the contribution of the information provided by network analysis on reproducibility, we also compare the reproducibility of subnetworks identified by MOBAS with that of individual associations. Table 3 shows the contingency tables comparing the distribution of individually significant genes on GAIN and WTCCC datasets and the genes that reside in the significant subnetworks. The four tables show in respective order the overlap between individually significant genes in GAIN and individually significant genes in WTCCC, individually significant genes in GAIN and the genes in a significant subnetwork identified on WTCCC, the genes in a significant subnetwork identified on GAIN and individually significant genes in WTCCC, and the genes in a significant subnetwork identified on GAIN and the genes in a significant subnetwork identified on WTCCC. While constructing this table, an FDR threshold of 0.05 is applied for a gene or subnetwork to be considered significant. Furthermore, a subnetwork is deemed significant only if it is significant according to both null models (permuted phenotype and permuted PPI network). We assess the significance of the association between any two variables in each table using chi-square test. As seen in the table, while the overlap between the two datasets is significant based on the individual association of genes with PS, the significance of the overlap is enhanced when network information is added. This observation suggests that MOBAS is able to recover genes that may not be individually associated with the disease according to one dataset but are functionally relevant and can exhibit significant individual association when other datasets are considered.

**Table 3:** The contingency between the individual genes and subnetworks identified on two independent psoriasis datasets

(a)		Significant in WTCCC	Not Significant in WTCCC
	Significant in GAIN	11	1
	Not significant in GAIN	28	2927
(b)		In significant WTCCC subnetwork	Not in significant WTCCC subnetwork
	Significant in GAIN	2	10
	Not significant in GAIN	13	2942
(c)		Significant in WTCCC	Not Significant in WTCCC
	In significant GAIN subnetwork	6	15
	Not in significant GAIN subnetwork	33	2913
(d)		In significant WTCCC subnetwork	Not in significant WTCCC subnetwork
	In significant GAIN subnetwork	13	8
	Not in significant GAIN subnetwork	2	2944

The *p* values of the contingency of each table according to chi-square test are (a) 1.72E-175, (b) 3.39E-15, (c) 4.32E-28, and (d) 0

#### Biological relevance

Several genome-wide linkage studies suggest that susceptibility loci for PS are clustered around the HLA region on chromosome 6p21 [41, 44]. The top subnetwork in both datasets includes HLA-B and HLA-C genes which are known to be associated with PS. Other HLA genes, KLRD1, B2M, and CD8A are also involved in immune response pathways and make the top subnetwork enriched in T cell-mediated immunity process (*p* value =8*e*−15). The second subnetwork also includes COL3A1 and PLCG1 which are involved in the immune system pathways.

### Robustness of the algorithm

**Fig. 6:**
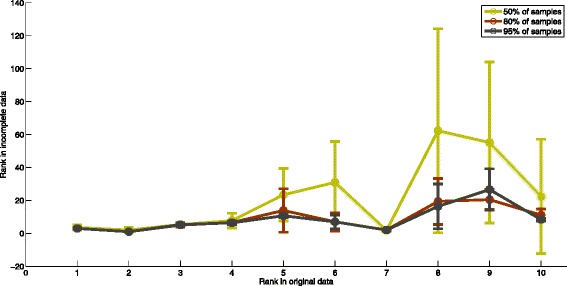
Robustness of MOBAS. The relation between the rank of the subnetwork in original data with rank of the subnetworks in incomplete data in ten different runs. Different colors represent different percentages of missing samples

The application of GWAS is not limited to identifying disease-associated variants, and the identification of variants associated with clinical variables such as response to treatment would be highly useful for personalized medicine. However, many studies may not be able to obtain genotype data for a sufficiently large number of samples. Therefore, designing an algorithm which is able to discover relevant associations even with smaller numbers of samples is important. In this section, we investigate whether our results deteriorate significantly with the lack of samples. For this purpose, we remove a fraction of the samples preserving the proportion between number of case samples and control samples. We remove 5, 15, and 50 % of samples of GAIN dataset randomly. We run the algorithm on the remaining samples and compare the top subnetworks identified in the incomplete data with the top subnetworks extracted from the entire set of samples. We repeat this analysis ten times with different sets of removed samples. Figure 6 shows the result of this analysis. The x axis shows the rank of subnetworks in original data. The y axis shows the rank of the subnetwork in incomplete data which has the maximum overlap with the original subnetworks. The error bar represents the standard deviation of the rank of subnetworks in ten different runs on incomplete data. As seen in the figure, the top subnetworks are also identified as top subnetworks on the incomplete data set. These results are promising in that even with half of the samples, we are able to identify subnetworks with high overlap with the significant subnetworks in original data.

## Conclusions

In this paper, with a view to facilitating the identification of disease-associated functional modules, we propose a novel methodology for scoring PPI subnetworks in terms of their association with a complex disease of interest and their network connectivity. Our experimental studies show that objective criteria for scoring subnetworks have to be selected carefully to ensure that the algorithms detect parsimonious subnetworks that are statistically significant and robust. In particular, we show that, with a carefully designed scoring scheme, network analysis is able to extract knowledge from GWAS data beyond the scope of the data itself. Namely, the subnetworks identified by the proposed method contain genes that do not exhibit significant association with the disease based on analysis of GWAS data but are known to have mechanistic role in the disease and are significant in the other dataset. Furthermore, the subnetworks identified by the proposed method include genes that are not yet reported to have a role in the disease, are not detected to be significant by GWAS, but have molecular functions that indicate potential involvement in the disease. Our results on psoriasis show that the subnetworks that MOBAS identified are reproducible between independent datasets.

The method presented in this paper focuses on a single network pattern: dense subgraphs of the PPI network. However, investigation of different network patterns may provide additional insights on the relationships between different disease-associated genes and molecular mechanisms of these associations.

## Endnote

^1^A preliminary version of this article appeared in the Proceedings of the 17th European Conference on Evolutionary Computation, Machine Learning and Data Mining in Computational Biology.

## Abbreviations

DAFM: disease-associated functional modules
dbGaP: database of genotypes and phenotypes
FDR: false discovery rate
GAIN: genetic association information network
GWAS: genome-wide association studies
MOBAS: modularity-based scoring
PPI: protein-protein interaction
PS: psoriasis
SNP: single nucleotide polymorphism
T2D: type II diabetes
WTCCC: Wellcome-Trust Case Control Consortium
